# Role of vitamin D deficiency in systemic lupus erythematosus incidence and aggravation

**DOI:** 10.1007/s13317-017-0101-x

**Published:** 2017-12-26

**Authors:** Tohid Hassanalilou, Leila Khalili, Saeid Ghavamzadeh, Ali Shokri, Laleh Payahoo, Yaser Khaje Bishak

**Affiliations:** 1 Department of Nutrition, Faculty of Nutrition and Food Sciences, Tabriz University of Medical Sciences, Tabriz, I.R. Iran; 2 Department of Human Nutrition, Medicine Faculty, Urmia University of Medical Sciences, Urmia, Iran; 3 Talented Student Center, Student Research Committee, Faculty of Nutrition and Food Sciences, Tabriz University of Medical Sciences, Tabriz, I.R. Iran

**Keywords:** Vitamin D deficiency, Systemic lupus erythematosus, SLE incidence, SLE aggravation

## Abstract

Vitamin D is one of the main groups of sterols; playing an important role in phospho-calcic metabolism. The conversion of 7-dehydrocholesterol to pre- vitamin D3 in the skin, through solar ultraviolet B radiation, is the main source of vitamin D. Since lupus patients are usually photosensitive, the risk of developing vitamin D deficiency in is high in this population. Although evidences showed the connotation between systemic lupus erythematosus (SLE) and vitamin D through which SLE can lead to lower vitamin D levels, it is also important to consider the possibility that vitamin D deficiency may have a causative role in SLE etiology. This paper analyzes existing data from various studies to highlight the role of vitamin D deficiency in SLE occurrence and aggravation and the probable efficacy of vitamin D supplementation on SLE patients. We searched “Science Direct” and “Pub Med” using “Vitamin D” and “SLE” for finding the studies focusing on the association between vitamin D deficiency and SLE incidence and consequences. Evidences show that vitamin D plays an important role in the pathogenesis and progression of SLE and vitamin D supplementation seems to ameliorate inflammatory and hemostatic markers; so, can improve clinical subsequent.

## Introduction

Systemic lupus erythematosus (SLE), a systemic autoimmune disease, can cause chronic inflammation and damage in several tissues and organs [1]. Genetic susceptibility and environmental factors are both responsible for the pathogenesis of SLE [2, 3]. Vitamin D deficiency is one of such factors [4]. Vitamin D plays vital role in mineral metabolism, and skeletal, cardiovascular and immune systems health [5]. The prevalence of vitamin D deficiency is high and evidence shows that it can contribute to the morbidity and mortality of numerous chronic diseases, including SLE [5]. As patients with SLE avoid the sun because of photosensitive rashes and potential for disease flare [5]; adequate vitamin D supplementation is vital for them. The vitamin D deficiency not only is known as a risk factor [4] of autoimmune diseases such as multiple sclerosis (MS) and type 1 diabetes (T1D) [6], but also can affect disease activity and disease damage in SLE patients [7]. This review is going to summarize the evidences about the effects of vitamin D deficiency in SLE occurrence and/or aggravation and its consequences.

## Vitamin D deficiency and SLE

Vitamin D, as a steroid hormone, exhibits regulatory effects on growth, proliferation, apoptosis and function of the immune system cells that are associated with pathophysiology of SLE [8]. Vitamin D inadequacy is highly prevalent in SLE patients due to the avoidance of sunshine, photoprotection, renal insufficiency and the use of medications such as glucocorticoids, anticonvulsants, antimalarials and the calcineurin inhibitors, which alter the metabolism of vitamin D or down regulate the functions of the vitamin D receptor [8]. Kamen et al. [5] found significantly lower serum 25-hydroxyvitamin D levels among recently diagnosed SLE patients compared to matched controls, and a high overall prevalence of vitamin D deficiency. The deficiency was seen in this population even in the summer, likely due to the use of sunscreens, avoidance of sun exposure, or darker skin pigment and the limited amount of vitamin D obtained from dietary sources [5]. The finding that African Americans and those with photosensitivity had the most severe vitamin D deficiency can be explained with this interpretation [5]. As found by Borba et al. [9] the level of 25OHD and 1,25(OH)2D3 in SLE patients with high activity was lower compared to patients with minimal activity and controls. Only one patient presented the desired 25OHD levels. The possible reason is decreased vitamin D production because of the lack of sunlight exposure, use of sunblock, or by the disease itself, like the deficiency observed in medical inpatients [10]. Increased metabolism or damaged 25-hydroxylation caused by drugs or even by the disease itself could be another explanation [9].

## Vitamin D deficiency and SLE incidence

**Fig. 1:**
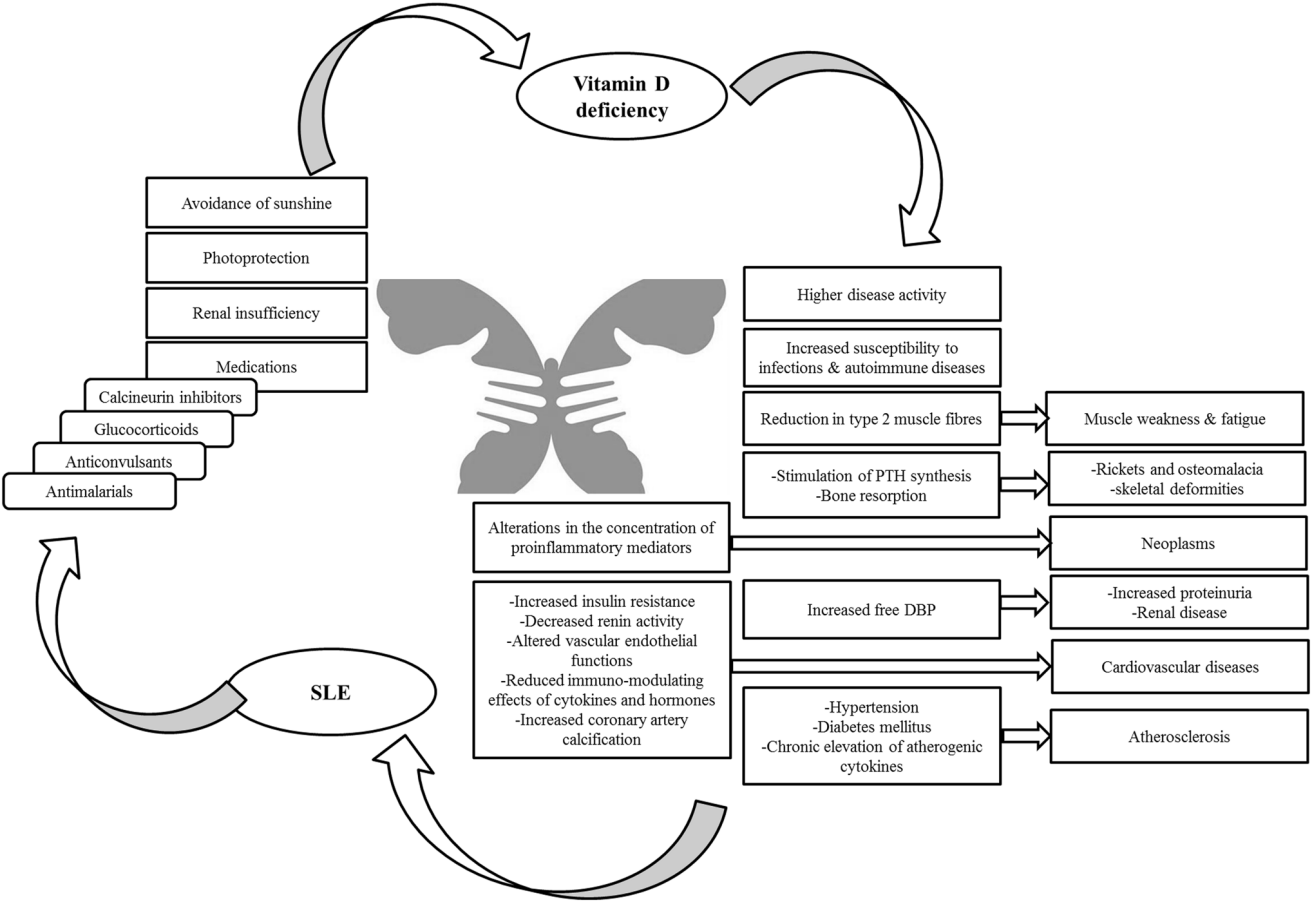
The two-sided relation between vitamin D and SLE showing that low levels of vitamin D resulted from SLE and SLE complications come from vitamin D deficiency

Vitamin D regulates the immune system by being involved in interleukin-2 (IL-2) inhibition, antibody production and in lymphocyte proliferation [11–13]. 1,25-dihydroxy vitamin D3 (1,25(OH)2 D3) inhibits IFN-ɣ secretion and by down-regulating NF-κB inversely controls IL-12 production [14]. When administered in vivo, 1,25(OH)2 D3 was found to have a preventative effect on autoimmune diseases, such as murine lupus [15]. Vitamin D deficiency is commonly reported in systemic lupus erythematosus [16]. The link between vitamin D and SLE is two sided (Fig. 1); so that, SLE may lead to lower vitamin D levels and vitamin D deficiency may have a causative role in SLE etiology and/or aggravation [6]. This perception is accumulating an important evidence base with regard to the matter that vitamin D deficiency is widely known as a risk factor of numerous autoimmune diseases, including MS and type 1 diabetes (T1D) [17]. By measuring serum vitamin D levels in individuals before MS onset, Munger et al. [18] showed that individuals with high 25(OH)D levels (100 nmol/L) have a 62% lower MS risk. In vitro studies showed that 1,25-dihydroxyvitamin D could prevent differentiation of dendritic cells and modulates T cell phenotype and function [19]. 1,25-dihydroxy vitamin D can inhibit T cell proliferation and cytokine production, inhibit proliferation of activated B cells, and impair generation of plasma cells [20, 21]. Differentiation of dendritic cells and subsequently production of type I interferon is [11] important in the pathogenesis of systemic lupus erythematosus (SLE) [22]. Therefore, by affecting immune system, vitamin D may play a preventive role in SLE incidence.

Establishing the temporal relationship between vitamin D deficiency and preceding disease onset is needed to determine a potentially causal role for vitamin D in SLE [6]. Disanto et al. [23] detected a clear seasonal distribution of beginnings for some of immune-related diseases, including MS and SLE, in which a peak in April and a trough exactly 6 months later in October were found. These findings implicate a varying seasonal factor such as UVB radiation and subsequent vitamin D synthesis in disease etiology. Considering the fact that the genes associated with SLE, MS, and T1D have been enriched for vitamin D receptor binding sites, it can be understood that vitamin D may possibly influence disease risk by regulating the SLE associated genes [24]. The immune modulating effect of vitamin D is established now; thus, it is logical that vitamin D deficiency is a risk factor, rather than a consequence of SLE [6].

Vitamin D activity is dependent on VDR (vitamin D receptor), a member of the nuclear hormone receptor superfamily. The VDR gene is located on chromosome 12q13.11 [25], and three polymorphisms, BsmI, ApaI (both in intron 8), and TaqI (in exon 9), have been identified at the 30-end of the gene [26]. As vitamin D presents immunosuppressive effects and there are potential link between vitamin D deficiency and autoimmune diseases, VDR polymorphisms that can affect VDR activity, have been evaluated as the probable cause of autoimmune diseases [24]. The meta-analysis, conducted by Lee et al. [27] addresses the link between VDR polymorphisms and RA and SLE susceptibility. According to the findings in addition to vitamin D deficiency, the vitamin D receptor (VDR) polymorphisms can confer susceptibility to immune-related diseases such as rheumatoid arthritis (RA) and systemic lupus erythematous (SLE) [27, 28].

## Vitamin D deficiency and SLE consequences

Low levels of vitamin D that is in relation with SLE activity, are mostly found in SLE patients [29–32]. In comparison with many other cells and tissues that harbor VDR, muscles are one of the most sensitive tissues that vitamin D deficiency causes weakness and fatigue [33, 34]. Moreover, the development of neoplasms and cardiovascular disease, increased susceptibility to infections, and autoimmune diseases are associated with vitamin D deficiency [33, 35].

## Vitamin D deficiency and disease activity

Considering the existence of VDRs on most cells of the immune system, the immunomodulatory effects of vitamin D can be understood [36]. The association between serum concentrations of vitamin D and the progression and development of autoimmune disorders has been focused on in several studies [37]. It has been revealed that disease activity and autoantibody production in SLE cases may be aggravated by vitamin D insufficiency [36]. Many of the immunomodulatory effects of vitamin D are opposite to the immunological abnormalities observed with disease activity in SLE patients [36]. So, it can be concluded that vitamin D deficiency is a risk factor for the onset and development of disease activity in SLE [36]. A cross-sectional study involving 378 patients with SLE from Europe to the Middle East [37] and the study conducted by Mok et al. [38] showed a significantly opposite relationship between 25-hydroxyvitamin D3 levels and disease activity scores. In another report, Amital et al. [37] revealed a significant inverse connection between the grade of SLE activity and serum vitamin D concentration. The results indicated that, among other factors, vitamin D deficiency can probably contribute to the progression of active disease in patients with SLE. In the study conducted on a large group of Australian patients with SLE, it had been shown that vitamin D insufficiency was associated with a higher disease activity and a rise in serum vitamin D level was associated with reduced disease activity over time [39]. Furthermore, vitamin D deficiency has been involved in the progression of rickets and osteomalacia, neoplasms and cardiovascular disease as well as in an increased susceptibility to infections and autoimmune diseases [33, 35].

## Vitamin D deficiency and fatigue and muscle strength

As shown in recent studies there is an inverse relationship between vitamin D levels and fatigue in SLE. Individuals with SLE have reduced exercise capacity [40], reduced strength [41], reduced quality of life [42] and elevated levels of fatigue [43]. More than 50% of people with SLE describe fatigue as the most disabling disease symptom [44]. Vitamin D receptors have been found on muscle cells [45, 46], which proves the theory of the direct effect of vitamin D on muscle tissue. Vitamin D deficiency can cause proximal muscle weakness with a reduction in type 2 muscle fibres [47]. Myositis in SLE is an uncommon but well defined condition with a reduction in strength, elevation in serum CK (creatine kinase) and abnormal muscle biopsy results [48]. In a study conducted by Stockton et al. [49] women in the SLE group were weaker in all muscle groups tested except shoulder abduction compared with the control group. The control group reported a median of 2.2 (range 1.0–3.8) on the FSS, which is representative of the reported predicted norm-based scores of 2.3 (0.7) [44]. In comparison, the SLE group reported significantly elevated fatigue of 5.1 (1.6–7.0), which is similar to the other published SLE cohorts [43, 50]. An observational study that conducted on 80 patients with SLE to evaluate the vitamin D insufficiency and deficiency, recommended oral vitamin D3 in patients with low baseline 25(OH)D levels [51]. The relationship between changes in 25(OH)D levels from baseline to changes in fatigue (measured by a 0–10 visual analog scale [VAS]) was analyzed. An inverse correlations was found between 25(OH)D levels and the VAS and between changes in 25(OH)D levels and changes in the VAS in patients with baseline 25(OH)D levels 30 ng/mL. It was recognized that increasing 25(OH)D levels may have a positive effect on fatigue. According to several researches dose adjustments should be based on monitoring of serum 25(OH)D levels [51]. In the general population, levels above 30 ng/mL are the recommended goal to avoid parathyroid hormone activation [33]. However, it is not clear whether these are the optimal levels in patients with lupus [51]. Ruiz-Irastorza et al. [51] recommended the use of dosages of vitamin D3 higher than 800 IU/day for SLE patients with vitamin D insufficiency or deficiency.

## Vitamin D deficiency and bone disease and fracture

Improving the intestinal absorption and renal resorption of calcium to increase its circulating levels that is mediated by the interactions between vitamin D and vitamin D receptors (VDRs) is the main metabolic effect of 1,25(OH)2D [33, 52]. Stimulation of PTH synthesis and bone resorption can be caused as the result of low levels of vitamin D. Continuance insufficiency of vitamin D results in rickets and osteomalacia, with skeletal deformities in children and bone pain and increased risk of fractures in adults [33]. The risk of osteoporosis and bone fractures is higher in patients with SLE [53]. Several factors are involved in the pathophysiology of bone disease in SLE, including environmental and hormonal factors, medications, inflammatory process, renal disease, and vitamin D deficiency as the result of photosensitivity [30, 32, 53–56]. Furthermore, alterations in the concentration of proinflammatory mediators have been described in SLE patients such as cytokines known by their potential role on bone metabolism [57, 58]. SLE patients are at high risk for fracture, even if with normal BMD values [9]. Recent meta-analyses showed that higher-dose vitamin D intake could reduce fall risk by 19% and fracture risk by 15–29% [59]. Considering the evidences, it is vital to evaluate vitamin D deficiency and to correct the vitamin D nutritional status in SLE subjects [9].

## Vitamin D deficiency and renal diseases

According to the researches there is a connotation between vitamin D deficiency and SLE renal disorders [32]. In a clinical trial conducted by Robinson et al. [60] serum 25(OH)D levels in patients with SLE were directly connected with serum albumin and inversely connected with the UP/C ratio and urinary DBP/C. A review of subjects with pediatric SLE revealed an association between proliferative SLE glomerulonephritis and vitamin D deficiency, and an average 10-ng/mL difference in serum 25(OH)D levels between subjects with and without nephritis [61]. Low serum 25(OH)D levels would decrease the amount of 25(OH)D bound to the DBP that is spilled in urine. DBP loss in urine may reflect the magnitude of proteinuria shown by SLE patients [60]. It has been found that a 20 ng/mL increase in vitamin D was associated with a 21% decrease in the probability of having a high activity score and a 15% decrease in the possibility of having clinically important proteinuria [62]. Studies on patients with stage 3 and 4 chronic kidney disease showed that the reduction in proteinuria in the paricalcitol (a vitamin D compound) treated patients was 3.2 times greater in comparison with the placebo group [63]. More researches are needed to evaluate the relationship among vitamin D deficiency in SLE patients and renal diseases.

## Vitamin D deficiency and cardiovascular diseases

As shown by evidences, vitamin D deficiency is associated with cardiovascular disease [38]. Observational studies have revealed a connotation between low 25(OH)D3 level and stroke, myocardial infarction, hypertensive heart disease, obesity, dyslipidemia, and diabetes mellitus [64–67]. Some of the suggested mechanisms for the adverse effects of vitamin D deficiency on the cardiovascular system include increased peripheral resistance to insulin, decreased renin activity, altered vascular endothelial functions, reduced immuno-modulating effects of cytokines and hormones such as IL-10, TNF-α, and PTH, and increased coronary artery calcification [68]. According to the third National Health and Nutrition Examination Survey (NHANES III) [69] patients within the lowest vitamin D level (20 ng/mL), had a significantly increased prevalence of selected CVD risk factors (including a history of diabetes and elevated blood pressure, fasting blood glucose, BMI and triglycerides) when compared with the highest level (37 ng/mL).

Due to the increased prevalence of the traditional risk factors such as hypertension, diabetes mellitus, and chronic elevation of atherogenic cytokines, patients with SLE are at risk of atherosclerosis [70]. A Recent research showed that hypovitaminosis D in SLE patients was associated with increased fasting glucose level [71]. Mok et al. [38] showed a significant inverse relationship between the levels of 25(OH)D3 and SLE disease activity scores. Vitamin D deficiency was connected with higher LDL cholesterol and lower HDL cholesterol, higher triglyceride level, the presence of aPLs, and premenopausal status. Similarly, Ezzat et al. [72] revealed that there is an association between lower 25(OH)D levels and increased CVD risk factors, as well as increased SLE disease activity and damage indices, also with the presence of proteinuria, low complement levels and steroid use. More studies are needed to understand whether vitamin D levels can predict the progression of subclinical atherosclerosis as measured by imaging markers as well as cardiovascular events in patients with lupus.

## Vitamin D deficiency and malignancy

It has been proved that the risk of developing many chronic illnesses especially common cancers would be decreased by vitamin D [16]. Serum levels of 25(OH)D lower than 20 ng/mL among the general population is connected with an increased risk of dying from colon, ovarian, breast, and prostate cancer [33]. As mentioned before Vitamin D deficiency that is common in SLE, has been associated with cardiovascular disease, diabetes mellitus, and certain forms of cancer [73]. Vitamin D and its metabolites decrease the incidence of several types of cancer by stimulating mutual adherence of cells, inhibiting tumor angiogenesis [74–76], and enhancing intercellular communication through gap junctions [77], so strengthening the inhibition of proliferation that results from tight physical contact with adjacent cells within a tissue [78]. The metabolites of vitamin D help to the maintenance of the normal calcium gradient in the colon epithelial crypts [79], and high serum levels of 25(OH)D are connected with decreased proliferation of noncancerous but high-risk epithelial cells in the colon [80]. 1,25(OH)2D inhibits mitosis of breast epithelial cells [81]. Pulsatile release of ionized calcium from intracellular stores, including the endoplasmic reticulum, induces terminal differentiation and apoptosis [82], and 1,25(OH)2D improves this release [83]. It has been shown that the administration of 1100 IU of vitamin D daily over 4 years will reduce all-cancer risk in postmenopausal females [84]. A meta-analysis of 18 randomized controlled trials showed that subjects randomized to vitamin D supplementation experienced fewer deaths in comparison with those randomized to placebo [85]. Further investigations are needed to determine if vitamin D supplements can prevent the incidence of certain cancers in SLE patients.

## Role of vitamin D supplementation in SLE improvement

**Fig. 2:**
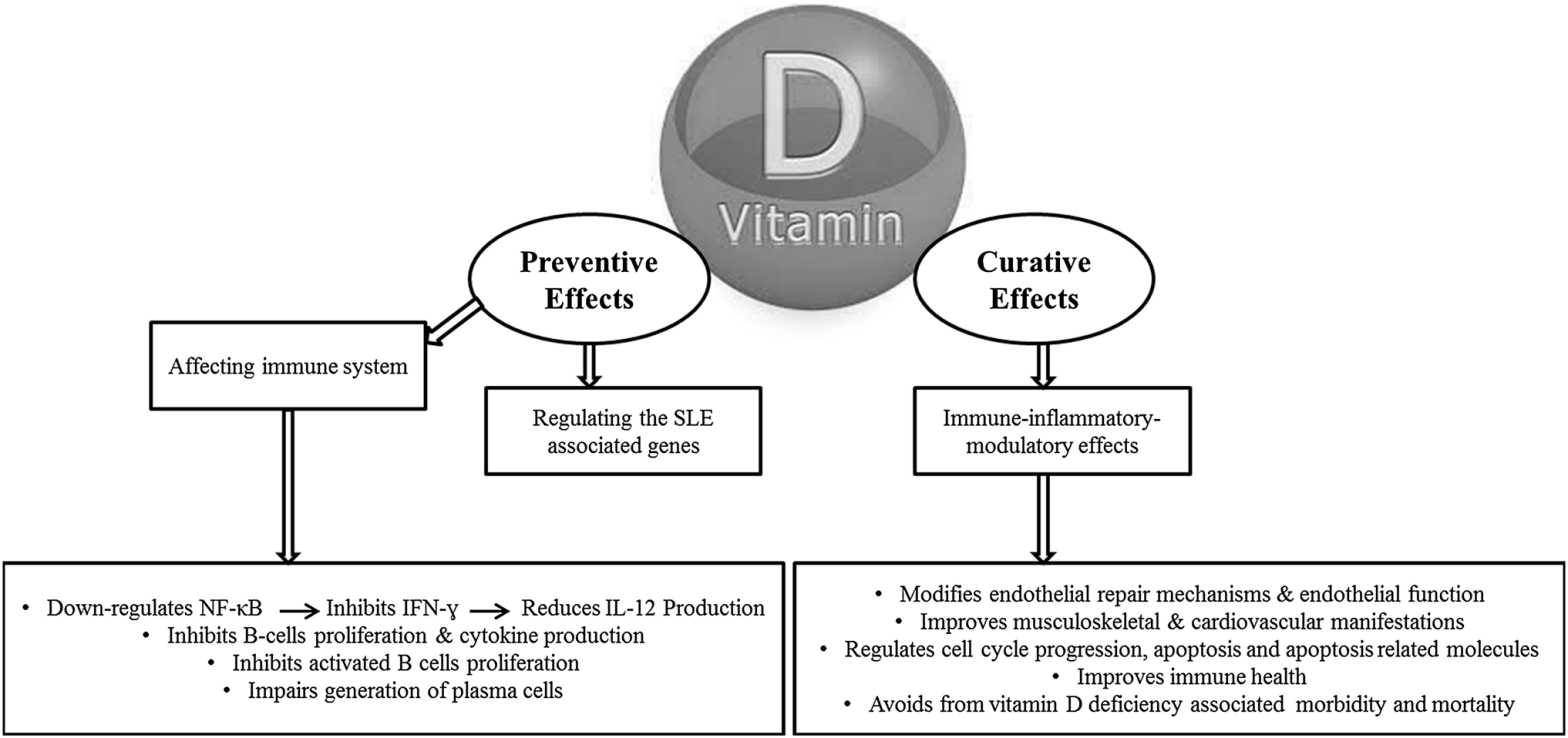
The beneficial effects of vitamin D in prevention and/or control of SLE

Vitamin D is a safe and inexpensive agent that is widely available. It could be beneficial as a disease suppressing intervention for SLE patients [5]. Besides its potential benefit in improvement of SLE activity, vitamin D is known to present immune-inflammatory-modulatory effect that can benefit musculoskeletal and cardiovascular manifestations of SLE. This role could also help maintain immune health; so, avoiding the excess vitamin D deficiency related morbidity and mortality [5] (Fig. 2). Recent evidences have shown the potential benefit of vitamin D supplementation in SLE patients (Table 1) [52, 62, 86–88]. Tabasi et al. [89] isolated peripheral blood mononuclear cells (PBMCs) from 25 SLE patients and cultured them in the presence of 50 nM of 1,25(OH)2D3. The results showed that Vitamin D has regulatory effects on cell cycle progression, apoptosis and apoptosis related molecules in lupus patients. The results of the investigation conducted by Reynolds et al. [90] demonstrate that vitamin D can positively modify endothelial repair mechanisms and so endothelial function in SLE patients that are susceptible for cardiovascular diseases. Abou-Raya et al. [87] showed an inverse association between 25(OH)D levels and disease activity markers. The observed that 25(OH)D levels were lowest among patients with active SLE. It was revealed that vitamin D deficiency could result in increased activity in SLE patients. Moreover, they found an improvement in the levels of proinflammatory cytokines after 12 months of vitamin D supplementation compared to placebo [87]. Early vitamin D supplementation in animal SLE models presented immunomodulatory effects [62] for instance dermatologic lesions, proteinuria, and anti-DNA were lesser in MRL/l mice supplemented with vitamin D [91].

**Table 1:** Studies about vitamin D supplementation in patients with SLE

Supplement	Study group	Effect	Side effect	References
100,000 IU of cholecalciferol per week for 4 weeks, followed by 100,000 IU of cholecalciferol per month for 6 months	20 SLE patients with hypovitaminosis D	Vitamin D was induced: a preferential increase of naïve CD4+ T cells, an increase of regulatory T cells a decrease of effector Th1 and Th17 cells a decrease of memory B cells and anti-DNA antibodies	Vitamin D was well tolerated	[86]
50,000 units of vitamin D-2 weekly plus 200 units of calcium/vitamin D-3 twice daily	1006 SLE patients with low levels of 25-hydroxyvitamin D (25[OH]D; < 40 ng/mL)	A 20-ng/mL increase in the 25(OH)D level was found that was associated with: A 21% decrease in the odds of having a high disease activity score and A 15% decrease in the odds of having clinically important proteinuria	Vitamin D was well tolerated	[62]
Oral cholecalciferol 2000 IU/day for 12 months	Patients with SLE and determined alterations in inflammatory and hemostatic markers and disease activity	At 12 months of therapy, there was a significant improvement in levels of inflammatory and hemostatic markers as well as disease activity in the treatment group	Vitamin D was well tolerated	[87]
Oral vitamin D3 for a median period of 24 months	Sixty patients with SLE	Inverse significant correlations between 25(OH)D levels and the VAS and between changes in 25(OH)D levels and changes in the VAS in patients with baseline 25(OH)D levels < 30 ng/mL were found	Vitamin D was well tolerated	[52]
Vitamin D supplementation for 6 months	SLE patients	The FoxP3^+^/IL-17A ratio in SLE patients after 6 months of vitamin D supplementation was higher than that in the baseline	Vitamin D was well tolerated	[88]

It should be noted that vitamin D supplementation could not always be completely safe. Vitamin D toxicity can cause by excessive oral supplementation [92]. The most important complications are hypercalciuria and hypercalcemia, however, hypercalcemia is mainly seen when the serum vitamin D levels reach 220 nmol/L and is most frequent when over 500 nmol/L [93] and the symptoms of hypercalcemia (nausea, vomiting, diarrhea, and headache) and renal stones appear in vitamin D intoxicated patients. It would be better to measure the baseline vitamin D level before supplementation. The Australian position statement on vitamin D in adults expresses that considering the individual variation of response to vitamin D supplementation, vitamin D levels are checked after 3 months [94]. Currently, there is no international consensus on the optimal dose for supplementation of vitamin D. European Food and Safety Authority recommends supplementation below 4000 IU/day [95]. Vitamin D supplementation in SLE patients is recommended as the increased vitamin D levels can ameliorate inflammatory and hemostatic markers and potentially clinical improvement [87]. Newly, ‘preventive’ treatment with vitamin D of subjects considered at high risk for developing autoimmune diseases has been suggested [28].

## Conclusion

Patients with SLE are at a clear risk of developing 25(OH)D deficiency because of photosensitivity and the frequently use of photoprotection [28]. In addition to the potential benefit of vitamin D replacement on SLE activity, patients will also avoid the excess morbidity and mortality associated with vitamin D deficiency [5]. More researches will help us better understand the role of vitamin D as immunomodulatory and determine the ideal range of serum 25(OH)D for musculoskeletal, cardiovascular, and immune health. Since vitamin D has an immune modulating effect, it is plausible that vitamin D deficiency is not only a risk factor, but also a consequence of SLE. According to several trials routine assessment of vitamin D levels and adequate supplementation of the vitamin in patients with SLE is recommended [5]. However, further large-scale studies are needed to establish the desired level of supplementation for prevention and/or improvement of SLE.
